# Ginsenoside Rd Attenuates Mitochondrial Permeability Transition and Cytochrome *c* Release in Isolated Spinal Cord Mitochondria: Involvement of Kinase-Mediated Pathways

**DOI:** 10.3390/ijms15069859

**Published:** 2014-06-03

**Authors:** Jin-Song Zhou, Jiang-Feng Wang, Bao-Rong He, Yong-Sheng Cui, Xiang-Yi Fang, Jian-Long Ni, Jie Chen, Kun-Zheng Wang

**Affiliations:** 1 Department of Orthopedics, the Second Affiliated Hospital of Medical School, Xi’an Jiaotong University, Xi’an 710004, China; E-Mails: zhoujs_xjtu@163.com (J.-S.Z.); nijl_xjtu@163.com (J.-L.N.); chenjie_xjtu@163.com (J.C.); 2 Department of Neurosurgery, the Second Affiliated Hospital of Shaanxi University of Chinese Medicine, Xi’an 712000, China; E-Mail: wangjf_xjtu@163.com; 3 Department of Orthopedics, Hong Hui Hospital, Xi’an Jiaotong University College of Medicine, Xi’an 710054, China; E-Mails: hebr_xjtu@163.com (B.-R.H.); fangxiang_xjtu@163.com (X.-Y.F.); 4 Department of Orthopedics, Shaanxi University of Chinese Medicine, Xi’an 712046, China; E-Mail: cuiys_xjtu@163.com; 5 Department of Orthopedics, Xi’an No. 5 Hospital, Xi’an 710082, China

**Keywords:** spinal cord injury, mitochondria, Ca^2+^, cytochrome *c*, protein kinases

## Abstract

Ginsenoside Rd (Rd), one of the main active ingredients in Panax ginseng, has multifunctional activity via different mechanisms and neuroprotective effects that are exerted probably via its antioxidant or free radical scavenger action. However, the effects of Rd on spinal cord mitochondrial dysfunction and underlying mechanisms are still obscure. In this study, we sought to investigate the *in vitro* effects of Rd on mitochondrial integrity and redox balance in isolated spinal cord mitochondria. We verified that Ca^2+^ dissipated the membrane potential, provoked mitochondrial swelling and decreased NAD(P)H matrix content, which were all attenuated by Rd pretreatment in a dose-dependent manner. In contrast, Rd was not able to inhibit Ca^2+^ induced mitochondrial hydrogen peroxide generation. The results of Western blot showed that Rd significantly increased the expression of p-Akt and p-ERK, but had no effects on phosphorylation of PKC and p38. In addition, Rd treatment significantly attenuated Ca^2+^ induced cytochrome *c* release, which was partly reversed by antagonists of Akt and ERK, but not p-38 inhibitor. The effects of bisindolylmaleimide, a PKC inhibitor, on Rd-induced inhibition of cytochrome *c* release seem to be at the level of its own detrimental activity on mitochondrial function. Furthermore, we also found that pretreatment with Rd *in vivo* (10 and 50 mg/kg) protected spinal cord mitochondria against Ca^2+^ induced mitochondrial membrane potential dissipation and cytochrome *c* release. It is concluded that Rd regulate mitochondrial permeability transition pore formation and cytochrome *c* release through protein kinases dependent mechanism involving activation of intramitochondrial Akt and ERK pathways.

## 1. Introduction

Paraplegia is defined as the impairment in motor or sensory function of the lower extremities. It is usually caused by traumatic or ischemic spinal cord injury that affects the neural elements in the thoracic, lumbar or sacral regions. It is reported that the incidence of spinal cord ischemia after thoracic and thoraco-abdominal aortic surgery is approximately 5%–8%, and there are more than 50 new traumatic spinal cord injury cases per million people each year [1,2]. The aetiology of spinal cord injury induced paraplegia is multi-factorial, and in the past few years various efforts to reduce this clinical problem have been made, including improving surgical technique [3,4], progress in monitoring and anaesthesia [5,6,7], and breakthrough in the prevention of complications [8,9]. However, there is still no effective treatment that leads to the full functional recovery of patients, whose deficits are generally permanent and irreversible [10,11,12].

Mitochondria are double-membraned organelles that primarily function as the powerhouses of cells through oxidative phosphorylation. Neuronal damage following spinal cord injury has been shown to be related to multiple intracellular changes, including toxic elevations of excitatory and inhibitory amino acids, neuronal edema, inflammation, vascular dysfunction, and activation of proteases, such as calpains and caspases, which are all associated with mitochondrial dysfunction in spinal cord neurons [13,14]. Inhibition or promotion of particular steps in the above mentioned molecular signaling cascades via targeting the entire organelle mitochondria might provide optimal neuronal protection or functional recovery following spinal cord injury. In support of this notion, mitochondria-targeted antioxidants, such as 5,5-dimethyl-pyrroline *N*-oxide, Mito-Q, and Mito-CP, have been shown to reduce oxidative damage and attenuate motor neuron degeneration in experimental spinal cord injury models [15]. Furthermore, targeting mitochondrial dysfunction in both primary insult and followed secondary injury events, many pharmacological agents, especially natural products extracted from medicinal plants, are suggested to have neuroprotective effects against traumatic and ischemic spinal cord injury [16,17,18].

Ginseng, the root of *Panax ginseng* C.A. Meyer, has been used as rejuvenating tonic for more than 2000 years in China [19]. In traditional Chinese medicine, it is known as the king of herbs because of the various pharmacological effects in the nervous system and cardiovascular system [20,21]. Ginsenosides are a special group of triterpenoid saponins that are found nearly exclusively in ginseng, and are considered to be responsible for most functions of ginseng. Previous studies have isolated more than 150 ginsenosides, with similar basic structure of a gonane steroid nucleus with 17 carbon atoms arranged in four rings [22,23]. Among the various ginsenosides, such as Rb, Rc, Rd, Re, Rf, and Rg, ginsenoside Rd (Rd) is one of the most abundant ingredients in the ginseng root and consequently has been accepted as one of the marker compounds of ginseng quality [24]. There is evidence that Rd exerts neuroprotective effects against excitotoxicity- and oxidative stress-induced injury in cultured neurons [25,26,27]. More recently, Rd was reported to ameliorate the histological and functional outcome after focal cerebral ischemia in rats [28,29,30,31]. However, the efficacy of Rd has not been established in animal models of spinal cord injury, and the molecular mechanism of Rd-induced neuroprotective activity has not been fully understood. In the present study, we sought to investigate the potential protective effects of Rd in isolated spinal cord mitochondria and the underlying mechanism with focus on mitochondrial permeability transition and cytochrome *c* release.

## 2. Results and Discussion

### 2.1. Rd (Ginsenoside Rd) Protects Isolated Spinal Cord Mitochondria against Ca^2+^ Induced Mitochondrial Membrane Potential Dissipation

**Figure 1:**
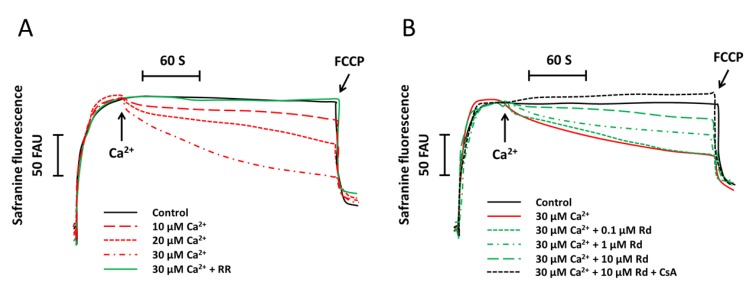
Rd (ginsenoside Rd) protects isolated spinal cord mitochondria against Ca^2+^ induced mitochondrial membrane potential dissipation. (**A**) Isolated spinal cord mitochondria (0.5 mg protein/mL supported by succinate) were treated with or without 1 μM ruthenium (RR) for 60 s before the incubation with increasing concentrations of Ca^2+^ (10, 20, or 30 μM). The mitochondrial membrane potential was measured up to 300 s; (**B**) Isolated spinal cord mitochondria (0.5 mg protein/mL supported by succinate) were treated with Rd at different concentrations (0.1, 1, or 10 μM) in the presence or absence of 1 μM cyclosporin A (CsA) for 60 s before the incubation with 30 μM Ca^2+^. The mitochondrial membrane potential was measured up to 300 s. Controls were performed in the absence of Ca^2+^, and 1 μM *p*-trifluoromethoxy-phenylhydrazone (FCCP) was added to induce maximal depolarization at the end of the measurements. Traces are representative of three independent experiments performed in duplicate and were expressed as fluorescence arbitrary units (FAU).

We first investigated the influence of increasing concentrations of Ca^2+^ (10–30 μM) on mitochondrial membrane potential in succinate supported spinal cord mitochondria. As shown in Figure 1A, Ca^2+^ treatment induced a dose-dependent increase of safranine fluorescence, which indicated the dissipation of mitochondrial membrane potential. It was also found that ruthenium (RR), an inhibitor of Ca^2+^ uptake by mitochondria, completely blocked the reduction of mitochondrial membrane potential, suggesting that the mitochondrial membrane depolarization was Ca^2+^ dependent. We next tested the effects of Rd on Ca^2+^ induced mitochondrial membrane depolarization. The results showed that Rd significantly attenuated the Ca^2+^ induced reduction of mitochondrial membrane potential in a dose-dependent fashion, although 0.1 μM Rd was not effective as compared with that in isolated mitochondria without Rd pretreatment (*p* > 0.05) (Figure 1B). Pretreatment with Rd plus cyclosporin A (CsA), a mitochondrial permeability transition (MPT) inhibitor, further prevented Ca^2+^ induced mitochondrial membrane depolarization as compared to Rd pretreatment alone.

### 2.2. Rd Protects Isolated Spinal Cord Mitochondria against Ca^2+^ Induced Mitochondrial Swelling

**Figure 2:**
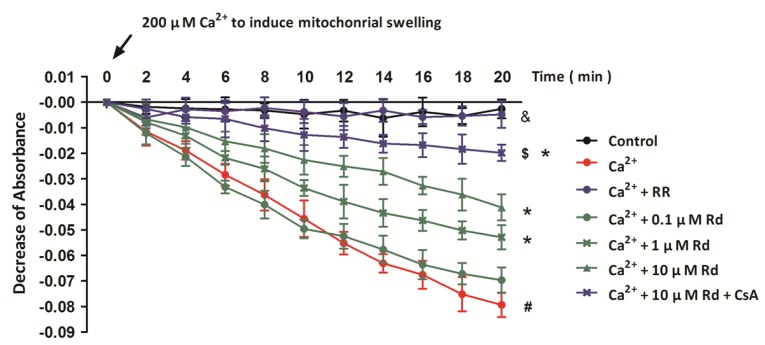
Rd protects isolated spinal cord mitochondria against Ca^2+^ induced mitochondrial swelling. Isolated spinal cord mitochondria (0.5 mg protein/mL supported by succinate) were treated with Rd at different concentrations (0.1, 1, or 10 μM) in the presence or absence of 1 μM RR and 1 μM CsA. Mitochondrial swelling was examined by monitoring the absorbance at 540 nm induced by 200 μM Ca^2+^. The control was measured without Ca^2+^. The data were represented as means ± SD from five experiments. ^#^ *p* < 0.05 *vs.* control. * *p* < 0.05 *vs.* Ca^2+^. ^$^ *p* < 0.05 *vs.* 10 μM + Ca^2+^. ^&^ *p* < 0.05 Ca^2+^ + RR *vs.* Ca^2+^.

To further verify the involvement of MPT inhibition in Rd induced inhibition of mitochondrial membrane depolarization after Ca^2+^ treatment, we also examined mitochondrial swelling in succinate supported spinal cord mitochondria. It has been shown previously that mitochondrial swelling is one of the common biomarkers of MPT pore opening, and in our *in vitro* model, 200 μM Ca^2+^ was added into the medium to induce mitochondrial swelling (Figure 2). We verified that 200 μM Ca^2+^ provoked extensive swelling in succinate supported spinal cord mitochondria, as represented by the significant decrease of absorbance at 540 nm. It was shown that RR fully prevented, whereas CsA attenuated Ca^2+^ induced mitochondrial swelling. The partial protective effect of CsA under our conditions is in accordance with the observation that CsA is less effective as MPT inhibitor in central nervous system than in isolated liver or heart mitochondria [32]. Furthermore, pretreatment with Rd at 1 or 10 μM in the medium partly reversed the Ca^2+^ induced mitochondrial swelling, but 0.1 μM Rd was not effective.

### 2.3. Effects of Rd on Mitochondrial H_2_O_2_ Generation and NAD(P)H Content after Ca^2+^ Treatment in Isolated Spinal Cord Mitochondria

**Figure 3:**
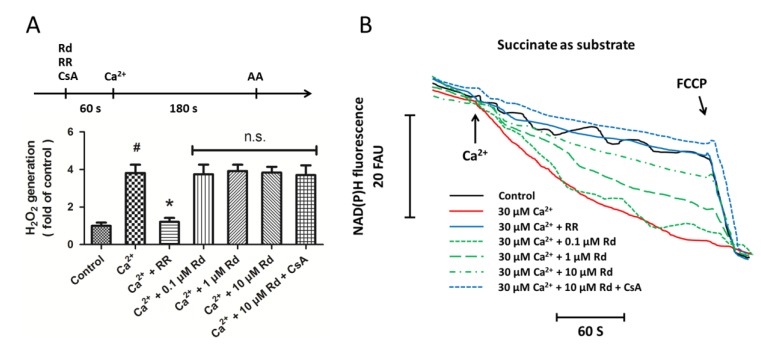
Effects of Rd on mitochondrial H_2_O_2_ generation and NAD(P)H content after Ca^2+^ treatment in isolated spinal cord mitochondria. (**A**) Isolated spinal cord mitochondria (0.5 mg protein/mL supported by succinate) were treated with Rd at different concentrations (0.1, 1, or 10 μM) in the presence or absence of 1 μM RR and 1 μM CsA. The mitochondrial H_2_O_2_ generation was measured, and 0.1 μg/mL Antimycin A (AA) was added to induce maximal H_2_O_2_ generation at the end of the measurements; (**B**) Isolated spinal cord mitochondria (0.5 mg protein/mL supported by succinate) were treated with Rd at different concentrations (0.1, 1, or 10 μM) in the presence or absence of 1 μM RR and 1 μM CsA. The mitochondrial NADH and NADPH content was measured, and 1 μM FCCP was added to induce maximal NAD(P)H oxidation at the end of the measurements. The data were represented as means ± SD from five experiments. Traces are representative of three independent experiments performed in duplicate and were expressed as fluorescence arbitrary units (FAU). ^#^ *p* < 0.05 *vs.* control. * *p* < 0.05 *vs.* Ca^2+^. n.s., not statistically significant.

In order to determine whether inhibition of reactive oxygen species (ROS) generation may be an underlying mechanism of Rd-induced protection in isolated spinal cord mitochondria, we detected the mitochondrial H_2_O_2_ generation under our *in vitro* experimental conditions. Figure 3A shows that Ca^2+^ significantly increased the mitochondrial H_2_O_2_ generation that were totally prevented by RR pretreatment. However, pretreatment with Rd at all concentrations used in our study did not alter H_2_O_2_ generation in succinate supported spinal cord mitochondria in the presence or absence of the MPT inhibitor CsA. These results suggest that H_2_O_2_ related ROS generation might not be involved in Rd induced protection against MPT pore formation. We also measured the influence of Rd and Ca^2+^ on NAD(P)H matrix content in succinate supported spinal cord mitochondria (Figure 3B). The results showed that Ca^2+^ addition significantly decreased the NAD(P)H content, which was fully prevented by RR pretreatment. Rd at 1 or 10 μM significantly attenuated the Ca^2+^ induced reduction of NAD(P)H content, and pretreatment with CsA further preserved the NAD(P)H content as compared to Rd alone.

### 2.4. Effects of Rd on the Phosphorylation of Mitochondrial Protein Kinases after Ca^2+^ Treatment in Isolated Spinal Cord Mitochondria

**Figure 4:**
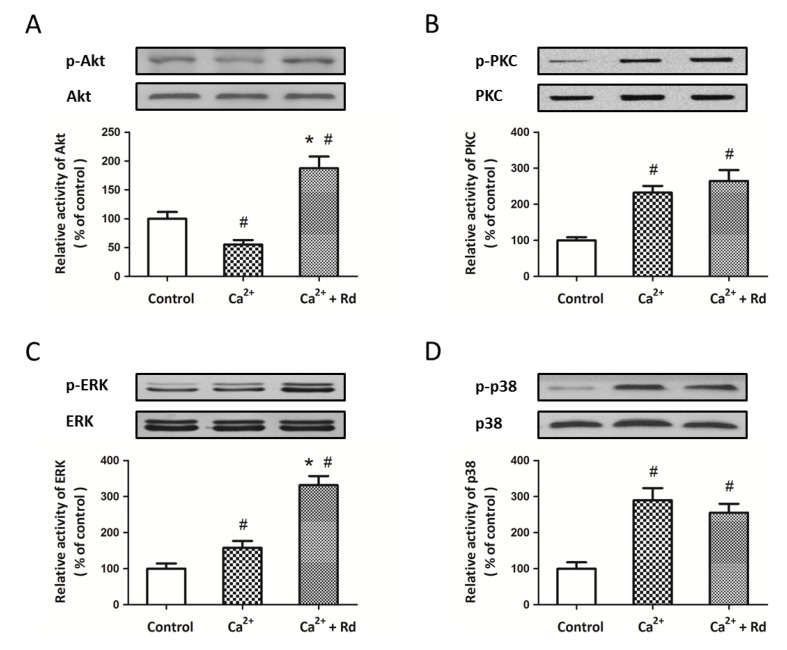
Effects of Rd on the phosphorylation of mitochondrial protein kinases after Ca^2+^ treatment in isolated spinal cord mitochondria. Isolated spinal cord mitochondria (0.5 mg protein/mL supported by succinate) were pretreated with 10 μM Rd for 10 min before the incubation with 30 μM Ca^2+^. The activation of Akt (**A**); PKC (**B**); ERK (**C**) and p38 (**D**) were detected by Western blotting analysis. The results were represented as the percentage of control. The data were represented as means ± SD from five experiments. ^#^ *p* < 0.05 *vs.* control. * *p* < 0.05 *vs.* Ca^2+^.

To elucidate signaling pathways underlying Rd induced protection in spinal cord mitochondria, we consider the possible involvement of mitochondrial kinases. Recent published studies have verified the presence of various protein kinases in mitochondria and their connection to MPT regulation [33,34]. We used Western blot analysis to detect the activation of Akt, PKC, ERK and p38 after Ca^2+^ treatment in the presence or absence of 10 μM Rd. As shown in Figure 4A, Ca^2+^ decreased the expression of p-Akt, whereas Rd pretreatment significantly increased the expression of p-Akt as compared to not only Ca^2+^ alone, but also control group. The results also showed that phosphorylation of ERK was increased by Ca^2+^ and further enhanced by Rd pretreatment (Figure 4C). As shown in Figure 4B and 4D, the expression of p-PKC and p-p38 were upregulated by Ca^2+^, but Rd pretreatment had no effect on p-PKC and p-p38 expression. In addition, neither Ca^2+^ nor Rd treatment had any effect on the total protein levels of Akt, PKC, ERK, and p-38.

### 2.5. Effects of Rd and Protein Kinase Inhibitors on Cytochrome c Release after Ca^2+^ Treatment in Isolated Spinal Cord Mitochondria

**Figure 5:**
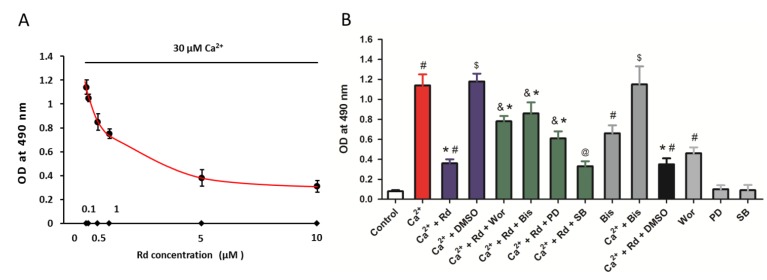
Effects of Rd and protein kinase inhibitors on cytochrome *c* release after Ca^2+^ treatment in isolated spinal cord mitochondria. (**A**) Isolated spinal cord mitochondria (0.5 mg protein/mL supported by succinate) were pretreated with Rd at different concentrations (0.1, 0.5, 1, 5, or 10 μM) for 10 min before the incubation with 30 μM Ca^2+^. The cytochrome *c* release induced by Ca^2+^ was measured; (**B**) Isolated spinal cord mitochondria (0.5 mg protein/mL supported by succinate) were pretreated with 1 μM wortmannin (Wor, PI_3_K inhibitor), 50 nM bisindolylmaleimide (Bis, PKC inhibitor), 10 μM PD98059 (PD, ERK inhibitor), or 10 μM SB202190 (SB, p38 inhibitor) in the presence or absence of 10 μM Rd for 10 min before the incubation with 30 μM Ca^2+^. The cytochrome *c* release induced by Ca^2+^ was measured. The data were represented as means ± SD from five experiments. ^#^ *p* < 0.05 *vs.* control. * *p* < 0.05 *vs.* Ca^2+^. ^&^ *p* < 0.05 *vs.* Ca^2+^ + Rd. ^@^ not statistically significant *vs.* Ca^2+^ + Rd. ^$^ not statistically significant *vs.* Ca^2+^.

MPT pore opening and followed cytochrome *c* release are facilitated by Ca^2+^ overload under various neuropathological conditions. Thus, we next detected the cytochrome *c* release in isolated spinal cord mitochondria after 30 μM Ca^2+^ treatment in the presence or absence of Rd at different concentrations (0.1, 0.5, 1, 5, or 10 μM) (Figure 5A). The results showed that Rd pretreatment inhibited cytochrome *c* release from mitochondria stimulated by Ca^2+^ in a dose-dependent manner. To further shed light on the relationship between the protection induced by Rd against mitochondrial cytochrome *c* release and its action on protein kinases regulation, the phosphorylation of Akt, PKC, ERK, and p38 were blocked, respectively, using the specific inhibitors, wortmannin (1 μM), bisindolylmaleimide (50 nM), PD98059 (10 μM), or SB202190 (10 μM). As shown in Figure 5B, the Rd induced reduction of mitochondrial cytochrome *c* release was partly reversed by Akt, PKC, and ERK inhibitors, but not p38 inhibitor. By using inhibitors alone, we found that treatment with Akt inhibitor alone increased the mitochondrial cytochrome *c* release, but PD98059 and SB 202190 had no effects on mitochondrial cytochrome *c* release. It was also observed that treatment with PKC inhibitor alone significantly increased the mitochondrial cytochrome *c* release, whereas bisindolylmaleimide could not further promote Ca^2+^ induced mitochondrial cytochrome *c* release.

### 2.6. Effects of Protein Kinase Inhibitors on Mitochondrial Membrane Potential Dissipation and Swelling after Ca^2+^ Treatment in Rd Pretreated Spinal Cord Mitochondria

**Figure 6:**
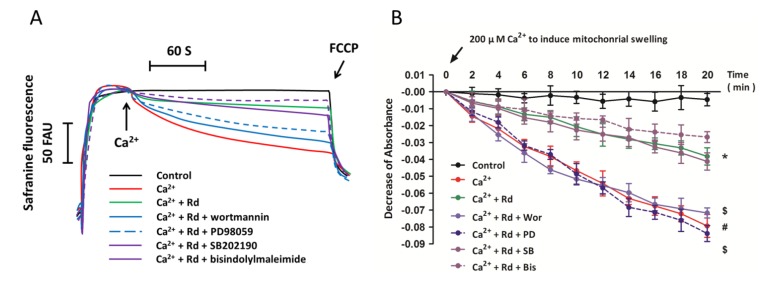
Effects of protein kinase inhibitors on mitochondrial membrane potential dissipation and swelling after Ca^2+^ treatment in Rd pretreated spinal cord mitochondria. Isolated spinal cord mitochondria (0.5 mg protein/mL supported by succinate) were pretreated with 1 μM wortmannin (Wor, PI_3_K inhibitor), 50 nM bisindolylmaleimide (Bis, PKC inhibitor), 10 μM PD98059 (PD, ERK inhibitor), or 10 μM SB202190 (SB, p38 inhibitor) in the presence or absence of 10 μM Rd for 10 min before the incubation with 30 μM Ca^2+^. (**A**) The mitochondrial membrane potential was measured up to 300 s. Controls were performed in the absence of Ca^2+^, and 1 μM FCCP was added to induce maximal depolarization at the end of the measurements. Traces are representative of three independent experiments performed in duplicate and were expressed as fluorescence arbitrary units (FAU); (**B**) Mitochondrial swelling was examined by monitoring the absorbance at 540 nm induced by 200 μM Ca^2+^. The control was measured without Ca^2+^. The data were represented as means ± SD from five experiments. ^#^ *p* < 0.05 *vs.* control. * *p* < 0.05 *vs.* Ca^2+^. ^$^ *p* < 0.05 *vs.* Ca^2+^ + Rd.

To further confirm the involvement of protein kinases in Rd-induced regulation of mitochondrial membrane potential dissipation and mitochondrial swelling, the phosphorylation of Akt, PKC, ERK, and p38 were blocked, respectively, using the specific inhibitors, wortmannin (Wor, 1 μM), bisindolylmaleimide (Bis, 50 nM), PD98059 (PD, 10 μM), or SB202190 (SB, 10 μM), and the mitochondrial membrane potential dissipation and mitochondrial swelling were measured. As shown in Figure 6A, Rd-induced attenuation of mitochondrial membrane potential dissipation was prevented by pretreatment with Akt and ERK inhibitors, but not PKC and p38 inhibitors. The results of mitochondrial swelling assay also showed that Akt and ERK inhibitors reversed the regulatory effects of Rd on Ca^2+^-induced mitochondrial swelling, but PKC and p38 inhibitors had not such effects.

### 2.7. Protective Activity of Rd against Mitochondrial Permeability Transition and Cytochrome c Release through in Vivo Administration

**Figure 7:**
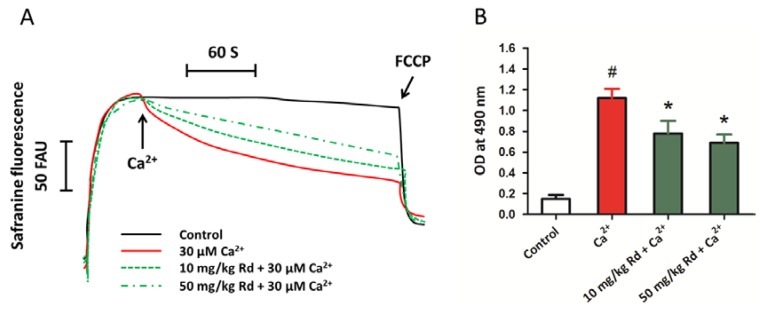
Protective activity of Rd against mitochondrial permeability transition and cytochrome *c* release through *in vivo* administration. Rats were pretreated with Rd at the concentrations of 10 or 50 mg/kg for 7 days before the spinal cord mitochondria were isolated, and then the isolated spinal cord mitochondria (0.5 mg protein/mL supported by succinate) were incubated with 30 μM Ca^2+^. The mitochondrial membrane potential was measured up to 300 s (**A**); and the cytochrome *c* release induced by Ca^2+^ was detected (**B**). The data were represented as means ± SD from five experiments. Traces are representative of three independent experiments performed in duplicate and were expressed as fluorescence arbitrary units (FAU). ^#^ *p* < 0.05 *vs.* control. * *p* < 0.05 *vs.* Ca^2+^.

To further confirm the protective effects of Rd in spinal cord tissues, we also determined mitochondrial membrane potential and mitochondrial cytochrome *c* release in isolated spinal cord mitochondria after *in vivo* administration of Rd at the dose of 10 and 50 mg/kg, which was chosen according to the previous studies [28,30]. The results showed that Rd pretreated spinal cord mitochondria were less vulnerable to Ca^2+^ induced mitochondrial membrane potential dissipation (Figure 7A). It was also found that pretreatment with Rd *in vivo* (both at the dose of 10 and 50 mg/kg) protected spinal cord mitochondria against Ca^2+^ induced cytochrome *c* release, although the effect was not dose-dependent as observed in *in vitro* experiments.

### 2.8. Discussion

Natural products found in herbs, fruits, vegetables, and plant extracts, are important parts of human health care in both developing and developed countries, with increasing commercial values. They are chemical compounds that usually have therapeutic activities for both treatable and incurable diseases, and many of the modern pharmaceutical industries have been developed on the basis of medicinal plants [35]. It is reported that more than 25% of the drugs prescribed at present come from medical plants, and approximately 60% of anti-infectious drugs on the market or under clinical trials are of natural origin [36]. Ginseng species have been used as rejuvenating tonic for more than 2000 years in China, and the protective effects of ginsenosides, the principle active ingredients, in central nervous system and cardiovascular system have been deeply investigated in rodents, dogs, and humans [20,24]. Ginsenoside Rd was shown to protect against oxidative stress and apoptotic cell death in several *in vitro* neuronal injury models, and attenuate neuronal damage in *in vivo* traumatic, ischemic or degenerative neurological diseases [24,25,26,29,30]. In the present study, we extended the protective effects of Rd to Ca^2+^ induced mitochondrial dysfunction in isolated spinal cord mitochondria, which could be involved in several related spinal cord related diseases, such as spinal cord ischemia and amyotrophic lateral sclerosis [37,38].

Although many compounds with antioxidant activities are demonstrated to have neuroprotective effects against mitochondrial dysfunction in spinal cord, Rd exerts unique advantages. Ginsenosides have already had thousands of years of human exposure with little reported toxicity [24]. Rd is a small lipophilic molecule that freely permeates through the phospholipids bilayers and interacts with various compounds to form different thiol-contained beneficial metabolites. In addition, the brain/serum ratio of Rd after intravenous injection was significantly higher than that of blood-brain barrier (BBB) impermeable albumin, even in energy deficient conditions [28,29], indicating an effective passage of Rd through the intact BBB. The mean plasma elimination half-life was approximately 14 h in mice, 24 h in dogs, and more than 19 h in healthy humans [39,40,41]. The relative long half-life as compared to other neuroprotective agents is useful for avoiding repeated dosing in patients. More importantly, Rd is being developed to treat patients with acute ischemic stroke, and a Phase III randomized, double-blind, placebocontrolled, multicenter clinical trial showed that Rd improves outcome of ischemic stroke in patients up to 90 days after stroke onset [42].

Most of the previous experiments on neuroprotective agents were performed in animals or using neuronal cell lines. In order to confirm the involvement of mitochondria and to elucidate the potential molecular mechanism underlying the protective activity of Rd, we used an *in vitro* system of isolated spinal cord mitochondria. Importantly, this system allowed us to investigate the roles of MPT, mitochondrial oxidative phosphorylation, and mitochondrial cytochrome *c* release in Rd induced beneficial effects and their cross talk, which might be an important supplement to the animal experiments. Our results showed that Ca^2+^ treatment induced mitochondrial membrane potential dissipation and significantly provoked mitochondrial swelling, which were fully prevented by RR, an inhibitor of the mitochondrial Ca^2+^ uniporter [43], but only partly attenuated by CsA. This is in accord with the data obtained in the isolated brain mitochondria where CsA failed to prevent Ca^2+^-induced depolarization or repolarize mitochondria when mitochondria were depolarized excessively [32]. It is emphasized that the MPT induction causes mitochondrial depolarization, organelle swelling, and dysregulation of metabolites, including Ca^2+^, Mg^2+^, glutathione, NAD(P)H, and H_2_O_2_ [44,45]. Interestingly, our data showed that Rd significantly attenuated Ca^2+^ induced reduction of NAD(P)H content in a dose-dependent manner, however, neither Rd treatment nor CsA had any effect on Ca^2+^ induced H_2_O_2_ generation in isolated mitochondria, indicating a H_2_O_2_ metabolism independent mechanism. This might be explained by that Rd could directly blocked the opening of MPT pore, thereby preserving NAD(P)H content and inhibiting cytochrome *c* release, with no effects on the H_2_O_2_ metabolism related enzymes, such as catalase (CAT) or superoxide dismutase (SOD) [46,47]. In accordance with our hypothesis, we found that Rd and CsA exerted synergetic effects on inhibition of Ca^2+^ induced mitochondrial cytochrome *c* release. In our *in vitro* conditions, MPT induction in the presence of Ca^2+^ seems to be a consequence of mitochondrial depolarization, since a significant decrease in membrane potential was observed. However, the exact mechanisms by which Rd affect the mitochondrial Ca^2+^ threshold for MPT opening, which makes spinal cord mitochondria less susceptible to insults, need to be further determined.

Proteins kinases are a group of kinase enzymes that modify other proteins through chemically adding phosphates to amino acid residues (phosphorylation), which result in a functional of the target substrate proteins by regulating enzymatic activity, subcellular location or interaction with other proteins [48]. Protein kinases mediate most of the signal transduction form the cell membrane to the nucleus in eukaryotic, but it is by no means the only pathway that signaling molecules can take. Numerous studies have shown that several proteins, such as Akt, PKC, and MAPKs [49,50], can translocated into the mitochondria to regulate phosphorylation of respiratory chain proteins, control the release of mitochondrial components, and thereby affect the whole cell responses [34]. We confirmed the expression and phosphorylation of Akt, PKC, ERK, and p38 in isolated spinal cord mitochondria in the present study, and found that their enzymatic activities were differently regulated by Ca^2+^ treatment, indicating the involvement of these kinases in MPT opening, cytochrome *c* release, and followed mitochondrial dysfunction. This point of view was further supported by that obvious increase of cytochrome *c* release was observed after treating isolated mitochondria with PKC inhibitor Bis alone, which was consistent with a recent study on purified liver mitochondria [51]. Many studies using pharmacologic inhibitors of MAPKs have demonstrated that ERK and p38 can modulate mitochondrial functions, especially those associated with cell death, and increased activation of Akt and PKC were also shown to exert pro-survival effects against mitochondrial related apoptotic cell death [52,53,54]. More recently, Rd was demonstrated to play protective roles via regulating PI3K/Akt and ERK pathways in both glutamate-induced excitotoxicity and myocardial ischemia/reperfusion injury [55,56]. Akt exerts its protective effects via phosphorylation of diverse target molecules (such as Bcl-2 family and GSK-3), preserving mitochondrial integrity [57,58]. A recent study showed that Rd attenuates myocardial ischemia/reperfusion injury via a Akt-mediated mitochondrial-dependent apoptotic pathway [56], which was also confirmed in transient forebrain ischemia models [59]. The role of ERK activation in the Ca^2+^ related neuronal death was disputable. Despite the volume of evidence supporting the p-ERK elevation after ischemic injury as detrimental effects that were essential for oxidative stress and inflammation-related cell death, numerous studies demonstrated that ERK activation contributed to protective effects of many neuroprotectants. For example, TGF-β1 blocked neuronal injuries by increasing ERK phosphorylation and the activated ERK pathway suppressed Bad protein levels and caspase-3 activation [60]. As a well-known neuroprotectant, estrogen also elicited rapid ERK activation against β-amyloid toxicity and quinolinic acid-induced cell death [61,62]. Data from our study might be another evidence for the protective role of ERK activation in Ca^2+^-induced mitochondrial dysfunction. Our present data using kinases inhibitors showed that Ca^2+^-induced MPT opening and cytochrome *c* release were partly reversed by Akt and ERK inhibitors, but not p38 inhibitor. Because Rd has no effects on PKC activation in isolated mitochondria as shown in Figure 4, the effects of Bis on Rd-induced protection (inhibition of cytochrome *c* release) seemed to be at the level of its own detrimental activity on mitochondrial function. All these data suggest that Rd attenuates Ca^2+^-induced mitochondrial dysfunction through different regulation of mitochondrial protein kinases, the targets of which might be different from those of cytosolic ones [34,51].

## 3. Experimental Section

### 3.1. Animals

We used male C57BL/6J mice (3-month old, 25–28 g). The animals had continuous access to food and water and were housed in cages in a room maintained at 20–22 °C with a 12 h light/12 h dark cycle. All experimental protocols and animal handling procedures were performed in accordance with the National Institutes of Health (NIH) guidelines for the use of experimental animals (NIH Publications No. 80-23, revised 1996). All efforts were made to minimize animal number and their suffering.

### 3.2. Mitochondria Purification

Mitochondria were isolated from the mouse spinal cord tissues and purification of their outer membranes was performed by differential ultracentrifugation. To prepare detergent lysates, the spinal cord tissues were frozen at −70 °C, thawed and treated with the lysing buffer with protease inhibitors cocktail for 2 h. The suspensions were centrifuged at 800× *g* for 10 min at 4 °C. The resulting lysate was cleared by centrifugation at 25,000× *g* for 20 min and dialyze. The final pellet was gently washed and resuspended in isolation buffer without ethyleneglycol bistetraacetic acid (EGTA), at an approximate protein concentration of 0.5 mg/mL. Total protein concentration was determined using Enhanced BCA Protein Assay Kit (Beijing Biosynthesis Biotechnology, Beijing, China).

### 3.3. Rd Treatment

Rd with a purity of 98% was obtained from Tai-He Biopharmaceutical Co. Ltd (Guangzhou, China). Stock solutions of Rd were prepared in saline containing 10% 1,3-propanediol (*v*/*v*). In *in vitro* experiments, isolated spinal cord mitochondria (0.5 mg protein/mL supported by succinate) were pretreated with Rd at different concentrations (0.1, 1, or 10 μM) for 60 s (for the measurement of mitochondrial membrane potential, mitochondrial swelling, H_2_O_2_ production and NAD(P)H content) or for 10 min (for the measurement of mitochondrial kinases expression) before the incubation with Ca^2+^. In *in vivo* experiments, Rd at the dose of 10 or 50 mg/kg was administered intraperitoneally twice a day for 3 days before mitochondria purification.

### 3.4. Mitochondrial Membrane Potential Measurement

The mitochondrial membrane potential was measured by using the cationic fluorescent probe safranine O as described previously [63]. The measurements were conducted on a temperature-controlled spectro-fluorometer with magnetic stirring operating at excitation and emission wavelengths of 495 and 586 nm, respectively. Mitochondrial preparations (0.5 mg protein/mL) were incubated at 37 °C with Rd for 60 s, and incubated with CaCl_2_ (10, 20, or 30 μM) afterward. In the end of the measurements, maximal depolarization was induced by 1 μM FCCP. The results were expressed as FAU.

### 3.5. Determination of Mitochondrial Swelling

Mitochondria swelling was measured following a previously published protocol [64]. Briefly, isolated mitochondria were suspended in fresh swelling buffer (0.2 M sucrose, 10 mM Tris-MOPS, pH 7.4, 5 mM succinate, 1 mM phosphate, 2 μM rotenone, and 1 μM EGTA-Tris, pH 7.4) at 0.5 mg/mL, and the swelling of mitochondria was monitored by a decrease in absorbance at 540 nm in the presence of CaCl_2_ (200 μM).

### 3.6. Measurement of H_2_O_2_ Production

Mitochondrial H_2_O_2_ production was assessed on a temperature-controlled spectro-fluorometer with magnetic stirring operating at excitation of 563 and emission of 587 nm by using 5 mM succinate plus 4 μM rotenone as substrate. Mitochondrial preparations (0.5 mg protein/mL) were incubated at 37 °C with Rd for 60 s, and incubated with CaCl_2_ (30 μM) afterward. Antimycin A (AA, 0.1 μg/mL) was added at the end of the measurements for maximal H_2_O_2_ generation. The results were represented as the fold of control.

### 3.7. Measurement of NAD(P)H Content

Matrix mitochondrial NAD(P)H content was measured on a temperature-controlled spectro-fluorometer with magnetic stirring operating at an excitation wavelengths of 366 nm and emission wavelength of 450 nm by using 5 mM succinate as substrate. Mitochondrial preparations (0.5 mg protein/mL) were incubated at 37 °C with Rd for 60 s, and incubated with CaCl_2_ (30 μM) afterward. In the end of the measurements, the maximal NAD(P)H oxidation was induced by 1 μM FCCP. The results were expressed as FAU.

### 3.8. Western Blot Analysis

Equivalent amounts of protein (40 μg per lane) were loaded and separated by 10% SDS-PAGE gels, and transferred to polyvinylidene difluoride (PVDF) membranes. Membranes were blocked with 5% nonfat milk solution in tris-buffered saline with 0.1% Triton X-100 (TBST) for 1 h, and then incubated overnight at 4 °C with the following primary antibody dilutions in TBST: anti-p-Akt (1:800), Akt (1:1000), p-PKC (1:500), PKC (1:1200), p-ERK (1:600), ERK (1:1000), p-p38 (1:500) and p38 (1:1200). After that the membranes were washed and incubated with secondary antibody for 1 h at room temperature. An analysis software named Image J was used to quantify the optical density of each band. The relative activity of Akt, PKC, ERK and p-38 were calculated by dividing the intensity of phosphorylated kinase bands by the intensity of the total kinase bands, and the data are presented as ratios to control values.

### 3.9. Determination of Cytochrome c Release

The purified mitochondria (0.5 mg protein/mL) were incubated with 30 μM CaCl_2_ in the presence or absence of Rd for 10 min at room temperature and were immediately pelleted by centrifugation (10 min, 7000× *g*) at 4 °C. To investigate the potential involvement of mitochondrial protein kinases, the kinases inhibitors (wortmannin, Wor, 1 μM; bisindolylmaleimide, Bis, 50 nM; PD98059, PD, 10 μM or SB202190, SB, 10 μM) were applied 10 min prior CaCl_2_ treatment. The mitochondria supernatants were collected and tested for the cytochrome *c* release by sandwich assay as described previously [65]. Experimental values of optical density (OD 490 nm) were calculated within the linear part of the calibration curve built with bovine cytochrome *c*.

### 3.10. Statistical Analysis

Statistical analysis was performed using SPSS 16.0, a statistical software package. Statistical evaluation of the data was performed by one-way analysis of variance (ANOVA) followed by Bonferroni’s multiple comparisons or unpaired *t* test (two groups). A value of *p* < 0.05 was considered statistically significant.

## 4. Conclusions

In conclusion, data from our study revealed that Rd protects isolated spinal cord mitochondria against Ca^2+^ induced MPT and cytochrome *c* release in a mitochondrial protein kinases-dependent manner. These effects of Rd in our *in vitro* system were mediated by the activation of Akt and ERK, but not related to the activities of PKC and p38 kinases. These results may implicate the possible therapeutic potential of Rd in the management of mitochondrial dysfunction in spinal cord injury, which needs to be further determined in clinical trials.
